# Corticosteroid therapy in refractory shock following cardiac arrest: a randomized, double-blind, placebo-controlled, trial

**DOI:** 10.1186/s13054-016-1257-x

**Published:** 2016-04-03

**Authors:** Michael W. Donnino, Lars W. Andersen, Katherine M. Berg, Maureen Chase, Robert Sherwin, Howard Smithline, Erin Carney, Long Ngo, Parth V. Patel, Xiaowen Liu, Donald Cutlip, Peter Zimetbaum, Michael N. Cocchi, the collaborating authors from the Beth Israel Deaconess Medical Center’s Center for Resuscitation Science Research Group

**Affiliations:** Department of Emergency Medicine, Beth Israel Deaconess Medical Center, Boston, MA USA; Department of Medicine, Division of Pulmonary and Critical Care, Beth Israel Deaconess Medical Center, Boston, MA USA; Department of Anesthesiology, Aarhus University Hospital, Aarhus, Denmark; Department of Emergency Medicine, Wayne State University School of Medicine, Detroit, MI USA; Department of Emergency Medicine, Baystate Medical Center, Springfield, MA USA; Shared Resources, Georgetown University Medical Center, Washington, DC USA; Department of Medicine, Division of General Medicine and Primary Care, Beth Israel Deaconess Medical Center, Boston, MA USA; Department of Cardiology, Beth Israel Deaconess Medical Center, Boston, MA USA; Department of Anesthesia Critical Care, Division of Critical Care, Beth Israel Deaconess Medical Center, Boston, MA USA; Research Center for Emergency Medicine, Aarhus University Hospital, Aarhus, Denmark

**Keywords:** Cardiac arrest, Steroids, Hydrocortisone, Shock, Vasopressors, Adrenal

## Abstract

**Background:**

The purpose of this study was to determine whether the provision of corticosteroids improves time to shock reversal and outcomes in patients with post-cardiac arrest shock.

**Methods:**

We conducted a randomized, double-blind trial of post-cardiac arrest patients in shock, defined as vasopressor support for a minimum of 1 hour. Patients were randomized to intravenous hydrocortisone 100 mg or placebo every 8 hours for 7 days or until shock reversal. The primary endpoint was time to shock reversal.

**Results:**

Fifty patients were included with 25 in each group. There was no difference in time to shock reversal between groups (hazard ratio: 0.83 [95 % CI: 0.40–1.75], *p* = 0.63). We found no difference in secondary outcomes including shock reversal (52 % vs. 60 %, *p* = 0.57), good neurological outcome (24 % vs. 32 %, *p* = 0.53) or survival to discharge (28 % vs. 36 %, *p* = 0.54) between the hydrocortisone and placebo groups. Of the patients with a baseline cortisol < 15 ug/dL, 100 % (6/6) in the hydrocortisone group achieved shock reversal compared to 33 % (1/3) in the placebo group (*p* = 0.08). All patients in the placebo group died (100 %; 3/3) whereas 50 % (3/6) died in the hydrocortisone group (*p* = 0.43).

**Conclusions:**

In a population of cardiac arrest patients with vasopressor-dependent shock, treatment with hydrocortisone did not improve time to shock reversal, rate of shock reversal, or clinical outcomes when compared to placebo.

**Clinical trial registration:**

Clinicaltrials.gov: NCT00676585, registration date: May 9, 2008.

## Background

Cardiac arrest occurs in over 400,000 patients in the United States each year [1], and the overall mortality for cardiac arrest remains dismal with a survival rate less than 10 % [1]. In an attempt to improve survival and quality of life, international cardiac arrest guidelines emphasize not only the importance of optimizing intra-arrest treatment, but also the management of patients during the post-cardiac arrest period [2–4]. Unfortunately, due to a paucity of studies investigating new medical treatments, clinicians have little to offer to post-cardiac arrest patients other than supportive care and temperature management [5].

Adrenal insufficiency is common in critical illness and post-cardiac arrest and is associated with poor outcome [6–12]. Adrenal insufficiency in the post-cardiac arrest state can be explained by multiple pathophysiological mechanisms including ischemia/reperfusion injury of the adrenal glands, increased metabolic demand, and the ongoing systemic inflammatory response after the cardiac arrest [13]. The relationship between adrenal insufficiency and hemodynamic compromise has been established in primary adrenal insufficiency. Corticosteroids, while controversial, are currently suggested in patients with refractory septic shock as a mean of reducing time to shock reversal and potentially improving mortality [14–16]. Previous studies have demonstrated multiple similarities between the post-cardiac arrest shock state and that of septic shock [12, 17–19]. Thus, a physiologic rationale exists for the potential efficacy of corticosteroid therapy in post-cardiac arrest patients with shock. Both the International Liaison Committee on Resuscitation (ILCOR) [20] and the American Heart Association (AHA) [5] state that the utility of corticosteroids remains unknown and that a critical knowledge gap exists in this area.

We hypothesized that the administration of hydrocortisone to post-cardiac arrest patients in refractory shock would improve hemodynamics leading to more rapid shock reversal and ultimately improvement in survival.

## Methods

### Study design

We conducted a multi-center, double-blind, randomized, placebo-controlled trial of hydrocortisone in post-cardiac arrest patients in shock. The study was conducted at Beth Israel Deaconess Medical Center (BIDMC), MA, USA (coordinating site), Wayne State Medical Center, MI, USA and Baystate Medical Center, MA, USA. The Committee on Clinical Investigations at BIDMC (2007-P-000227), Wayne State University Human Investigation Committee (082108MP2F) and the Institutional Review Board at Baystate Medical Center (132387) approved the study. The trial was registered at clinicaltrials.gov (NCT00676585). All patients were consented and enrolled via legal authorized representatives. Drs. Donnino and Andersen had full access to all the data in the study and take responsibility for the integrity of the data and the accuracy of the data analysis.

### Patients and intervention

The emergency department and intensive care units were screened for eligible patients between January 2008 and March 2014. We included patients if they met all of the following criteria: age ≥ 18 years, out-of-hospital or in-hospital cardiac arrest, and post-cardiac arrest vasopressor dependence for at least 1 hour. We defined vasopressor dependence as a continuous infusion of norepinephrine, dopamine (≥5 mcg/kg/min), vasopressin (>0.04 units/min), phenylephrine and/or epinephrine. Dobutamine or other inotropes at any dose were not considered vasopressor dependence. We excluded patients if they met one or more of the following criteria: (1) vasopressor dependence before the cardiac arrest, (2) chronic use of steroids prior to the cardiac arrest, (3) clinical indication for steroids or provision of steroids by the clinical team, (4) “do-not-resuscitate” (DNR) or “comfort measures only” (CMO) designation prior to enrollment, and (5) inability to obtain consent.

Patients were randomized in a 1:1 ratio to either hydrocortisone or placebo. The intervention consisted of intravenous hydrocortisone 100 mg every 8 hours for a total of 7 days or until 24 hours after shock reversal. The placebo was identical in appearance to the active treatment and patients, healthcare personnel and the research team remained blinded throughout the study period.

### Outcomes and data collection

The primary outcome was time to shock reversal defined as at least 24 hours off all vasopressor medications [21]. Secondary outcomes included shock reversal (yes/no), cytokine levels (see below), the cumulative vasopressor dose within 24 hours, and mortality and good neurological outcome at hospital discharge. Neurological outcome was defined using the cerebral performance category (CPC) scale, which ranges from 1 (normal) to 5 (brain death) [22]. We considered a CPC of 1 or 2 a “good neurologic outcome” and a CPC of 3, 4 or 5 and death a “poor neurologic outcome” consistent with previous cardiac arrest investigations [23–25]. The cumulative vasopressor dose was calculated as the total dose of vasopressors within the first 24 hours after study drug administration. Since different vasopressors have different potency we used the following formula: cumulative vasopressor dose (μg/kg) = norepinephrine (μg/kg) + dopamine/2 (μg/kg) + epinephrine (μg/kg) + phenylephrine/10 (μg/kg) as previously used [26–28].

### Cosyntropin test and cytokines

An adrenocorticotropic hormone (cosyntropin) stimulation test was performed immediately before the first dose of the study medication: cosyntropin (250 μg) was administered immediately after an initial blood draw and then blood was drawn again 30 and 60 minutes thereafter. Cortisol levels were measured in all these samples. After the 60-minute draw, the study medication was administered. Adrenal insufficiency was defined in two ways: absolute: baseline cortisol < 15 ug/dL and relative: < 9 ug/dL increase at 30 minutes or 60 minutes (using the highest value) after the cosyntropin test as compared to baseline, consistent with previous literature.

Additional blood draws for cytokine measurements were obtained 24 hours after the study drug administration. Plasma samples were analyzed for interleukin (IL)-6 and IL-10 using a customized Meso Scale Discovery (MSD) Human Multiplex Panel (Rockville, MD, USA) according to the manufacturer’s protocol. IL-6 and IL-10 are measured in pg/mL.

### Sample size and statistical analysis

We determined that a sample size of 50 patients (25 per group) would yield 86 % power based on the following assumptions: the survival distributions followed an exponential function and the median time to event for the placebo group was 33 hours and 13 hours for the hydrocortisone group with a follow-up period of 7 days. The type I error rate was set at 0.05 and we used a two-sided test with the Lakatos normal approximation for the log-rank test.

The study population was characterized using descriptive statistics; categorical variables are provided as counts with frequencies and continuous variables as means with standard deviations (SD) or medians with quartiles depending on the normality of the data.

The primary endpoint of time to shock reversal was complicated by a high incidence of death prior to reversal of shock. To account for this we classified death as a competing risk event and used the estimated cumulative incidence function (CIF) to illustrate the comparison of CIFs between the two treatment groups. These estimated CIF functions were derived from the estimation of the Fine-Gray competing risk model [29]. We tested the sub-distribution hazards of these two CIFs and obtained the estimated hazard ratio with 95 % confidence intervals.

Given that cytokine values were severely right-skewed, we log-transformed them before analysis and then compared values between the groups with a *t* test. Differences between groups (hydrocortisone vs. placebo) in other outcomes were evaluated using Fisher’s exact test for categorical variables, and two-sample *t* tests or Wilcoxon rank-sum tests for continuous variables.

Preplanned subgroup analyses were performed in those with baseline absolute and relative adrenal insufficiency. All hypothesis tests were two-sided, with a significance level of *p* < 0.05. We performed no adjustment for multiple comparisons and all secondary analysis should therefore be considered exploratory. Statistical analyses were conducted with the use of SAS software, version 9.4 (SAS Institute Inc., Cary, NC, USA).

## Results

### Patient characteristics

Fifty patients were included; 25 received hydrocortisone and 25 received placebo. Forty-eight patients were enrolled at the coordinating site. No patients were lost to follow-up and no patients had the study drug stopped prematurely or required unblinding. The mean age for the complete study population was 69 (SD: 14) years and 17 (34 %) were female. The majority of patients (38 [76 %]) had an out-of-hospital cardiac arrest and 34 (68 %) died before hospital discharge. Baseline characteristics for each group are presented in Table 1. The groups were well matched on baseline characteristics except for a higher frequency of hypertension in the placebo group and a higher frequency of renal disease in the hydrocortisone group.

**Table 1:** Selected baseline characteristics of the study patients^a^

Characteristic	Hydrocortisone (n = 25)	Placebo (n = 25)
Demographics		
Age (years)	71 (13)	66 (15)
Sex (female)	9 (36)	8 (32)
Race (white)	21 (84)	18 (78)
Co-morbidities		
Coronary artery disease	11 (44)	9 (36)
Congestive heart failure	6 (24)	8 (32)
Hyperlipidemia	7 (28)	9 (36)
Hypertension	11 (44)	19 (76)
Chronic obstructive pulmonary disease	2 (8)	1 (4)
Diabetes	5 (20)	6 (24)
Liver disease	0 (0)	0 (0)
Renal disease	6 (24)	2 (8)
Cancer	2 (8)	1 (4)
Location of the arrest		
In-hospital	6 (24)	6 (24)
Out-of-hospital	19 (76)	19 (76)
Arrest characteristics		
Initial rhythm (shockable)	9 (36)	10 (40)
Downtime (minutes)	21 (10, 30)	16 (10, 38)
Presumed cause of the arrest		
Primary cardiac	13 (52)	12 (48)
Pulmonary embolism	1 (4)	1 (4)
Respiratory	5 (20)	6 (24)
Metabolic/electrolyte	2 (8)	1 (4)
Sepsis	1 (4)	0 (0)
Other/unknown	3 (12)	5 (20)
Vital signs at enrollment		
Heart rate	84 (18)	82 (21)
Systolic blood pressure	103 (97, 132)	109 (93, 130)
Diastolic blood pressure	61 (51, 70)	59 (48, 67)
Respiratory rate	22 (5)	23 (5)
Glasgow coma scale	3 (3, 6)	3 (3, 4)
Laboratory values at enrollment		
Lactate (mmol/L)	2.8 (1.9, 5.3)	3.9 (1.9, 6.5)
Glucose (mg/dL)	237 (108)	226 (99)
pH	7.25 (0.14)	7.24 (0.13)
Time from ROSC to study drug (hours)	9.9 (7.3, 19.6)	12.7 (7.8, 15.6)
Time from start of vasopressor(s) to study drug (hours)	9.7 (6.3, 18.0)	11.4 (7.5, 15.0)
APACHE II score at enrollment	29 (5)	30 (7)
Induced hypothermia	19 (76)	16 (64)

*ROSC* return of spontaneous circulation, *APACHE* Acute Physiology and Chronic Health Evaluation

^a^Categorical variables are presented as count (frequency) and continues variables as mean (standard deviation) or median (quartiles) depending on the normality of the data. Data missing on one patient for downtime, one patient for Glasgow coma score, one patient for pH, one patient for lactate, two patients for glucose and one patient on time from start of vasopressors to study drug

### Clinical and safety outcomes

There was no difference in the primary outcome of time to shock reversal between the groups (hazard ratio: 0.83 [95 % CI: 0.40–1.75], *p* = 0.63). There was no difference in shock reversal (13 [52 %] vs. 15 [60 %], *p* = 0.78), good neurological outcome (6 [24 %] vs. 8 [32 %], *p* = 0.75) or survival to discharge (7 [28 %] vs. 9 [36 %], *p* = 0.76) between the hydrocortisone and placebo groups. Three (12 %) patients in the hydrocortisone group and four (16 %) in the placebo group died within 24 hours of study drug administration (*p* = 0.68). There was no difference between the two groups in the mode of death (*p* = 0.92, Table 2). The cumulative vasopressor dose within the first 24 hours was calculated on 47 patients with available data (24 in the hydrocortisone group and 23 in the placebo group). There was no difference in the cumulative vasopressor dose between the hydrocortisone and placebo groups (842 μg/kg [418, 3120] vs. 437 μg/kg [170, 4237], *p* = 0.62) during the first 24 hours. In patients who achieved shock reversal (i.e., excluding those who died while on vasopressors), there was no difference in the time to shock reversal between the hydrocortisone and placebo groups (55 hours [30, 59] vs. 49 [25, 71] hours, *p* = 0.86).

**Table 2:** Mode of death

	Hydrocortisone (n = 18)	Placebo (n = 16)
Refractory shock	1 (6)	1 (6)
Sudden cardiac arrest	2 (11)	2 (13)
Co-morbid disease withdrawal of care	3 (17)	1 (6)
Primary neurological withdrawal of care	12 (67)	11 (69)
Other/unknown	0 (0)	1 (6)

Potential adverse events and selected laboratory values are presented in Table 3. There was no difference in bleeding, administration of new antibiotics or administration of an insulin infusion between the hydrocortisone and placebo groups. Glucose and sodium levels were similar between the two treatment groups at 6, 24, 48, and 72 hours after initiation of study drug. There was no difference in the white blood cell count between groups at 6 or 24 hours, however patients who received hydrocortisone had higher white blood cell count at 48 and 72 hours after administration of the study drug.

**Table 3:** Potential adverse events and selected laboratory values^a^

	Hydrocortisone (n = 25)	Placebo (n = 25)	*p* value
Adverse event after study drug			
Any bleeding	5 (20 %)	2 (9 %)	0.42
New or changed antibiotics	9 (36 %)	11 (44 %)	0.77
New insulin infusion	7 (28 %)	6 (24 %)	1.00
Laboratory values			
Glucose level (mg/dL)			
6 hour	242 (151)	230 (118)	0.76
24 hour	218 (109)	199 (90)	0.54
48 hour	163 (50)	155 (59)	0.66
72 hour	150 (52)	144 (61)	0.79
Sodium level (mmol/L)			
6 hour	139 (5)	139 (6)	0.78
24 hour	139 (5)	138 (5)	0.37
48 hour	139 (7)	139 (5)	0.80
72 hour	143 (6)	139 (8)	0.09
White blood count (x 10^3^)			
6 hour	15.0 (6.9)	17.0 (6.7)	0.43
24 hour	17.8 (8.7)	15.5 (4.7)	0.29
48 hour	21.5 (9.3)	12.6 (3.4)	<0.001
72 hour	19.2 (8.8)	11.9 (2.9)	0.005

^a^Categorical variables are presented as count (frequency) and continues variables as mean (standard deviation)

### Cytokines

**Fig. 1:**
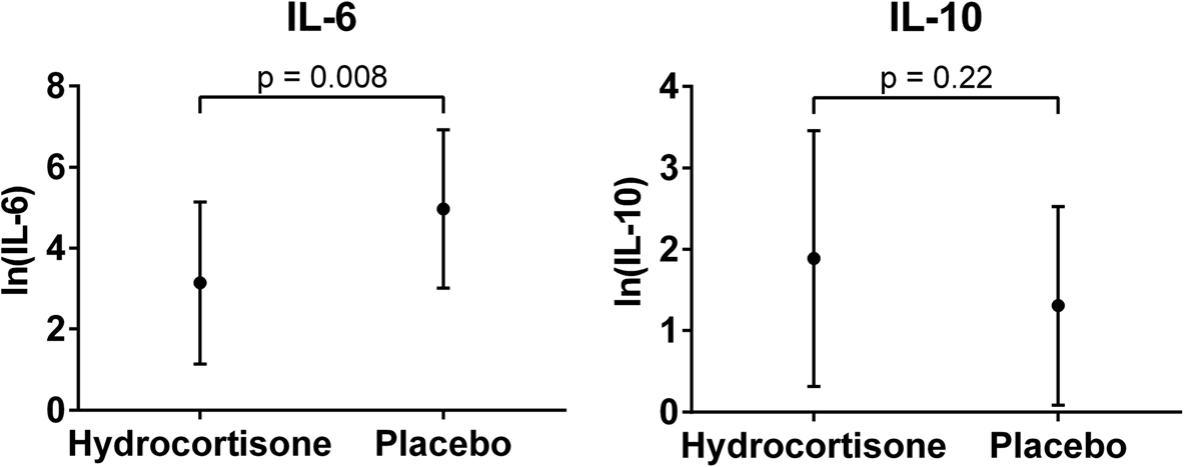
Cytokine levels at 24 hours. Log-transformed interleukin (IL)-6 (*left*) and IL-10 (*right*) levels 24 hours after study drug administration. There was significantly lower IL-6 levels at 24 hours in the hydrocortisone group compared to the placebo group (*p* = 0.008) but no difference in IL-10 levels (*p* = 0.22). The *dots* indicate the means and the *error bars* the standard deviation

Thirty-seven patients had cytokine levels measured at the 24-hour time point and were included in the analysis; 19 in the hydrocortisone group and 18 in the placebo group. Patients in the hydrocortisone group had significantly lower log-transformed IL-6 levels at the 24-hour time point as compared to the placebo group (3.14 [SD: 2.00] vs. 4.97 [SD: 1.96], *p* = 0.008, Fig. 1). There was no difference in log-transformed IL-10 levels (1.89 [SD: 1.57] vs. 1.31 [SD: 1.22], *p* = 0.22, Fig. 1) at the 24-hour time point.

### Patients with adrenal insufficiency

Baseline cortisol levels were available on 47 patients and 46 had follow-up cortisol levels. Nine patients had baseline absolute cortisol deficiency (six in the hydrocortisone group and three in the placebo group). The sample size in this subgroup was too small to perform the competing risk analysis. All six patients (100 %) in the hydrocortisone group had shock reversal as compared to one patient (33 %) in the placebo group (*p* = 0.08). There was no difference in the proportion of patients with good neurological outcome (2 [33 %] vs. 0 [0 %], *p* = 0.50) or survival to hospital discharge (3 [50 %] vs. 0 [0 %], *p* = 0.46) between the hydrocortisone and placebo groups.

Twenty-one patients had relative adrenal insufficiency (11 in the hydrocortisone group and 10 in the placebo group). There was no difference in shock reversal (3 [27 %] vs. 6 [60 %], *p* = 0.20), good neurological outcome (1 [18 %] vs. 4 [20 %], *p* = 0.36), or survival to hospital discharge (3 [27 %] vs. 4 [40 %], *p* = 0.66) between the hydrocortisone and placebo groups in this subgroup of patients.

## Discussion

In this study, we found no difference in time to shock reversal or other clinical outcomes in post-cardiac arrest patients with shock receiving hydrocortisone compared to placebo. While the corticosteroid group displayed increased white cell counts at 48 and 72 hours, we did not detect any significant differences in the side effect profiles between the groups. To the best of our knowledge, this is the first randomized trial to specifically evaluate the efficacy of hydrocortisone in the post-cardiac arrest patient population [5, 20].

The post-cardiac arrest syndrome is characterized by a variety of pathophysiological features similar to septic shock states including a systemic inflammatory response and hemodynamic perturbations, which may include microcirculatory dysfunction and myocardial suppression [17, 19, 30, 31]. Corticosteroid therapy in this setting could work via both immunologic and hemodynamic mechanisms. Corticosteroids are known to modulate the systemic inflammatory response [32, 33] and animal models have shown preserved myocardial function and improvement of arterial reactivity with the administration of physiological doses of corticosteroids [34, 35]. Despite the physiological rationale for corticosteroid therapy in this patient population and the fact that the utility of corticosteroids have been relatively well studied in the setting of septic shock [36], there is a lack of research on the efficacy of corticosteroids in post-cardiac arrest patients [5, 20].

A recent study by Mentzelopoulos et al. examined the effects of corticosteroids and vasopressin during and after in-hospital cardiac arrest. In this study, patients who received vasopressin and methylprednisolone during the cardiac arrest and hydrocortisone in the post-arrest period (if in shock) had improved clinical outcomes [37]. There are multiple potential explanations for the differences between studies. First, and most importantly, the study by Mentzelopoulos et al. included multiple interventions in the treatment arm both during and after the cardiac arrest. As such, it is difficult to make firm conclusions about any specific interventions [38]. Second, the patient populations differ substantially between studies (for example, we enrolled 76 % out-of-hospital arrest patients whereas Mentzelopoulos et al. enrolled only inpatients). Lastly, the sample size in the current study may have been too small to detect a significant difference between groups. In light of our findings, we believe that the provision of post-cardiac arrest corticosteroids in the context of the Mentzelopoulos et al. trial protocol would need to be specifically evaluated even if the full protocol were validated.

Multiple studies have examined adrenal insufficiency in the post-cardiac arrest population [8–12]. Although some inconsistency exists between these studies, they have generally found that impaired adrenal function is common [8] and that both low baseline cortisol levels [9, 10] and impaired cortisol response to adrenocorticotropic hormone stimulation are associated with poor outcomes [11, 12]. One study found that cortisol levels are rarely checked in patients with vasopressor-dependent shock post-cardiac arrest [8]. Out of a total group of 69 patients in that study, cortisol levels were only measured in nine (13 %) and corticosteroids were provided for the indication of shock in 12 (17 %) [8]. In the current study, we found that nine (18 %) of included patients had absolute baseline cortisol deficiency (<15 ug/dL) and 21 (42 %) had relative adrenal insufficiency. Although we did not find any significant differences in outcomes between those receiving hydrocortisone and placebo in these subgroups, we did see a nonsignificant difference in shock reversal in those with absolute adrenal insufficiency with six patients (100 %) in the hydrocortisone group having shock reversal as compared to one patient (33 %) in the placebo group (*p* = 0.08). Future studies may determine whether these patients could benefit from corticosteroid therapy.

Corticosteroids are well known to modulate the immune system and could theoretically have an effect on the post-cardiac arrest inflammatory response. In our study, we found that IL-6 levels were decreased in the hydrocortisone group as compared to the placebo group at 24 hours. We and others have previously reported that increased IL-6 levels measured after cardiac arrest are associated with increased mortality and worse neurological outcome [39–41]. While hydrocortisone attenuated IL-6 levels, there were no associated differences in clinical outcomes. These findings raise the possibility that IL-6 elevation after arrest may be an epiphenomenon and not necessarily in the causal pathway of persistent or ongoing injury. For reasons that remain unclear, we did not find any difference between groups in IL-10 levels, though IL-10 is considered to be an anti-inflammatory cytokine.

Our study has several limitations. First, the heterogeneity of our study population may not have allowed for assessment of subsets of patients that would be more or less likely to benefit from corticosteroids, or may have led to inclusion of population for which the ultimate outcome was less modifiable given baseline injury severity. Second, we found that the majority of patients succumbed to death from neurological causes and not hemodynamic compromise. In contrast, corticosteroids would theoretically be most efficacious in a population that has a higher burden of death from cardiovascular causes (i.e., refractory hemodynamic shock). That stated, we did not find that hydrocortisone led to a more rapid shock reversal or increased rate of shock reversal in this trial. Third, the numbers of patients with either relative or absolute adrenal insufficiency was too small for definitive conclusions in this important subgroup. Future study is necessary to help identify the value of corticosteroids in the deficient population, and our data suggests that a reasonable target population might be patients with a baseline cortisol < 15 ug/dL as opposed to those who fail to increase cortisol levels by 9 ug/dl with a cosyntropin stimulation test. The timing of corticosteroids may influence outcome, and this intervention could theoretically be beneficial earlier (i.e., just after return of spontaneous circulation in order to prevent subsequent injury) or later (i.e., after 24 hours hypothetically in the subset with adrenal exhaustion). Differing dosages, duration, or ways of providing (i.e., continuous versus intermittent infusion) may have impacted the findings. Finally, although three sites were enrolling patients, the vast majority of patients (48) were enrolled at the coordinating site.

## Conclusions

Hydrocortisone compared to placebo did not decrease time to shock reversal or improve overall shock reversal in post-cardiac arrest patients in shock.

## Key messages

- Until now, the effectiveness of corticosteroids in post-cardiac arrest patients with vasopressor-dependent shock is unknown
- We conducted a randomized, double-blind, placebo-controlled pilot trial of corticosteroids in post-cardiac arrest patients in shock
- In the overall population of patients with shock post-cardiac arrest, corticosteroids did not improve shock reversal or other clinical outcomes
- For the overall population of patients with shock post-cardiac arrest, corticosteroids did attenuate inflammation as represented by interleukin-6 levels
- For the subpopulation of patients with baseline adrenal insufficiency, corticosteroids had a near-significant improvement in shock reversal compared to placebo, though future studies with larger number of patients will be needed to evaluate this population.

## Abbreviations

CIF: cumulative incidence function
CPC: cerebral performance category
IL: interleukin
SD: standard deviation
